# Targeting mTOR in Pancreatic Ductal Adenocarcinoma

**DOI:** 10.3389/fonc.2016.00099

**Published:** 2016-04-25

**Authors:** Sentia Iriana, Shahzad Ahmed, Jun Gong, Alagappan Anand Annamalai, Richard Tuli, Andrew Eugene Hendifar

**Affiliations:** ^1^Department of Medicine, Cedars-Sinai Medical Center, Los Angeles, CA, USA; ^2^Department of Medical Oncology, City of Hope National Medical Center, Duarte, CA, USA; ^3^Department of Surgery, Cedars-Sinai Medical Center, Los Angeles, CA, USA; ^4^Department of Radiation Oncology, Cedars-Sinai Medical Center, Los Angeles, CA, USA; ^5^Department of Medicine, Division of Medical Oncology, Cedars-Sinai Medical Center, Los Angeles, CA, USA

**Keywords:** pancreatic ductal adenocarcinoma, mTOR, KRAS, preclinical, clinical trials

## Abstract

Treatment options for advanced pancreatic ductal adenocarcinoma (PDAC) are limited; however, new therapies targeting specific tumor-related molecular characteristics may help certain patient cohorts. Emerging preclinical data have shown that inhibition of mammalian target of rapamycin (mTOR) in specific KRAS-dependent PDAC subtypes leads to inhibition of tumorigenesis *in vitro* and *in vivo*. Early phase II studies of mono-mTOR inhibition have not shown promise. However, studies have shown that combined inhibition of multiple steps along the mTOR signaling pathway may lead to sustained responses by targeting mechanisms of tumor resistance. Coordinated inhibition of mTOR along with specific KRAS-dependent mutations in molecularly defined PDAC subpopulations may offer a viable alternative for treatment in the future.

## Introduction

### Epidemiology

Pancreatic ductal adenocarcinoma (PDAC) remains the fourth leading cause of cancer-related mortality throughout the United States, with 12.3 new cases reported per 100,000 men and women annually and 10.9 deaths reported per 100,000 men and women annually (1). The lifetime risk of developing pancreatic cancer is approximately 1.5% among men and women, with about 6.7% survival rate 5 years from diagnosis (1).

Pancreatic ductal adenocarcinoma is associated with poor prognosis. This is related to lack of standardized preventive screening, advanced age during diagnosis with median age being 71 years old, and advanced stage during diagnosis which has allowed only 15–20% to be surgically resectable at the time of presentation (2). These factors, and the aggressive nature of PDAC, has significantly limited the success of current treatment options, yielding continued low survival rates even in cases which are amenable to surgical resection, modest responses to chemotherapy and radiation, as well as development of resistance to such therapies (2, 3).

### Standards of Treatment

Resectability of PDAC is determined clinically by the patients underlying comorbidities and ability to tolerate major surgery, and radiographically based on involvement of the surrounding major vasculature including the superior mesenteric vein and artery, portal vein, celiac artery, and its branches, including the hepatic artery (2, 4). For resectable disease (stage I or II), pancreaticoduodenectomy is performed for tumors involving the head and uncinate of the pancreas, whereas distal pancreatectomy is performed for tumors of the body and tail and are considered potentially curative (2). However, only 15–20% of the patients are considered surgical candidates with many of them found to have microscopically positive margins (R1) discovered after surgery on final pathological review (2, 5), and others are considered medically unfit often secondary to malnutrition or advanced age and would not benefit from surgical resection or may experience major complications after pancreatic resection (6, 7).

Adjuvant (postoperative) therapy with the intention of reducing locoregional and metastatic recurrence has been shown to improve survival compared to postoperative observation alone (6). The Charité Onkologie (CONKO)-001 trial and the European Study Group for Pancreatic Cancer (ESPAC)-3 trial have investigated and established gemcitabine or 5-fluorouracil (FU) as effective avenues for adjuvant chemotherapy compared to observation (6). Neoadjuvant and adjuvant combination chemotherapy including fluorouracil, irinotecan, oxaliplatin, and leucovorin (FOLFIRINOX) and gemcitabine plus albumin-bound paclitaxel particles (nab-paclitaxel) is still being investigated, and adjuvant radiation has demonstrated mixed results (3, 6).

Stage III disease is divided into borderline resectable disease [<180° contact with the superior mesenteric artery (SMA) and locally advanced, unresectable disease (>180° contact with SMA)] (6). Neoadjuvant (preoperative) therapy is often recommended for borderline resectable disease to address the high probability of positive margins at resection (6). The combination of FOLFIRINOX and gemcitabine + nab-paclitaxel for the treatment of Stage III locally advanced unresectable tumors has limited data, but is commonly used. Again, adjuvant radiation is not adequately supported (2).

Curative resection is not recommended in stage IV PDAC, and treatment focuses on palliation. The use of FOLFIRINOX or gemcitabine + nab-paclitaxel has been shown to extend survival by at least 2 years in at least 10% of the patients, survival numbers that were rarely seen before and now represent standard first-line options, particularly in patients with good performance status [ECOG 0–1 (2, 8)]. Overall, multidisciplinary, symptomatic, and supportive therapies play an integral role in management (6).

### Rationale for mTOR Pathway

The investigation of genetic and molecular characteristics of PDAC remains a focus of current innovations in an effort to identify potential therapeutic targets. Genomic analysis of PDAC has revealed complex mutational patterns including near ubiquitious activation of KRAS, inactivation of >50% of major genetic pathways, such as TP53, SMAD4, and CDKN2A, involvement of 10% of genes in chromatin modification and DNA damage repair, and a collection of infrequently mutated genes that contribute to heterogeneity and create challenges in the development of targeted therapies (9). Affected gene mutations known to be important in pancreatic cancer turmorigenesis include TP52, SMAD4, CDKN2A, ARID1A, and ROBO2. In one evaluation, utilizing whole genome sequencing and copy number variation of PDAC, combining structural variation events with deleterious point mutations increased the prevalence of inactivation events to 74% for TP53, 31% for SMAD4, and 35% for CDKN2A (9).

The KRAS proto-oncogene is mutated in 90% of PDAC, with somatic alterations and locally rearranged, focal amplifications being quite common (6, 9, 10). Single point mutations in codon 12, 13, 59, or 61 of exon 2 and exon 3 of the KRAS oncogene impair intrinsic GTPase activity of KRAS and lead to a permanent active KRAS signaling pathway, resulting in proliferation and survival of cells (11). Mutation in KRAS leads to the uncontrolled activation of downstream intracellular signaling pathways such as the RAF/MEK/extracellular signal regulated kinase (ERK) and AKT contributing to tumor cell proliferation and survival (6, 12).

While wild-type KRAS has been described as a predictive marker for treatment success of EGFR, inhibitors such as erlotinib or cetuximab and panitumumab in metastatic non-small cell lung and colorectal cancer, its predictive and prognostic value in PDAC has not been clearly established (13). Inhibitors of KRAS have been largely unsuccessful in clinical trials and emphasis has been placed on its downstream pathways (6).

Additional downstream players of KRAS include phosphatidylinositol 3-kinase (PI3K) and AKT, which link ligation of growth factor receptors to the phosphorylation and activation of the serine/threonine kinase, mammalian target of rapamycin (mTOR), further downstream (14, 15). This downstream effector plays a role in cell survival, growth, proliferation, and motility, as well as a regulation of apoptosis (14, 15). mTOR exists as two complexes: mTORC1 that is rapamycin sensitive and mTORC2 that is largely rapamycin insensitive. mTORC1 interacts with the accessory protein Raptor-to-phosphorylate effectors S6 kinase 1, which ultimately enhances the translation of mRNAs, including ribosomal proteins, elongation factors, and insulin growth factor 2 (14). mTORC1 also phosphorylates 4EBP1 promoting dissociation of eIF4E from 4EBP1, thus relieving the inhibitory effect of 4EBP1 on eIF4E-dependent translation initiation, which again ultimately leads to increased translation of mRNAs (14). mTORC2 interacts with its companion RICTOR to phosphorylate PKC alpha and AKT contributing to cell survival, migration, and regulation of the actin cytoskeleton (14).

The mTOR complex is also closely related to the insulin/IGF-1 pathway. Decrease of adenosine triphosphate (ATP) production by metformin, for example, leads to AMPK activation and disruption of insulin/IGF-1 signaling through inhibition of mammalian target of rapamycin (8, 16). Inhibition of mTOR signaling, in turn, results in decreased protein synthesis and cell growth. Metformin can also inhibit mTOR signaling through activation of AMPK-independent pathways, including Rag GTPase (17) and REDD1 (18). AMPK-induced activation of tumor suppressor 53 (p53) and subsequent cell cycle arrest represents another potential mechanism of action of metformin in pancreatic cancer models (19). Clearly, the mTOR pathway is a key player in many biological processes including cell growth, regulation of actin cytoskeleton, gene transcription, ribosome biogenesis, mRNA translation, and cell survival and proliferation (14).

Upstream of mTOR, the PI3K/AKT pathway is influenced by PTEN, the negative regulator of PI3K signaling, which decreases its expression in many cancers including pancreatic, and may be downregulated through several mechanisms including mutation, deletion, and methylation (15).

Clinically investigated mTOR inhibitors include rapamycin (sirolimus) and its analogs, such as temsirolimus, everolimus, and ridaforolimus. These analogs combine with mTOR accessory protein FKBP12 forming complexes that bind to mTOR and inhibit mTORC1 downstream signaling, preventing S6K1 and 4EBP1 phosphorylation (14). While FKBP12-rapamycin complex cannot bind directly to mTORC2, prolonged treatments can disturb mTORC2 assembly and inhibit the phosphorylation of its downstream substrate AKT (14). However, inhibition of mTORC1 without mTORC2 inhibition may stimulate tyrosine kinase activity leading to AKT upregulation, a feedback loop that has been thought to contribute to mTOR resistance. Compared to rapamycin and its analogs, agents that were able to inhibit mTORC1 and mTORC2 were more effective in preclinical evaluations (20). These inhibitors have already been demonstrated to be promising therapeutic agents in other types of malignancies. A high response rate was observed with everolimus in Phase II trials in Hodgkin lymphoma, non-Hodgkin’s lymphoma, and breast cancer, and temsirolimus in Phase II/III trials in endometrial cancer and mantle-cell lymphoma (14).

## Preclinical Studies

### *In Vitro* mTOR Inhibitor Studies

*In vitro* studies have demonstrated diverse effects of mTOR inhibition on cell cycle arrest, autophagy, decreased desmoplastic inflammation, and inhibited epithelial-to-mesenchymal transition in preclinical studies of pancreatic cancer (Table 1). Rapamycin-induced autophagy and apoptosis in BxPC3, Su86.86, HPAF, Capan-1, and HS700T PDAC cell lines that correlated to cell line-specific levels of mTOR activity (21). Novel agents Alisertib and Plumbagin, induced cell cycle arrest, promoted autophagy, and inhibited epithelial-to-mesenchymal transition in PANC-1 and BxPC3 cell lines through inhibition of PI3K/AKT/mTOR signaling (22, 23).

**Table 1:** **The preclinical development of mTOR inhibitors in pancreatic cancer**.

Study agent	Source in which antitumor activity was demonstrated	Reference
Rapamycin	BxPC3, Su86.86, HS700T, HPAF, and Capan-1 cells (*in vitro*)	(21)
Rapamycin	PANC-1 cells (*in vitro*)	(24)
INK-128	Primary human PDAC, PANC-1, and MIA PaCa-2 cells (*in vitro*)	(25)
BEZ235 ± PD0325901, PKI-587 ± PD0325901, and GDC-0980 ± PD0325901	MIA PaCa-2 and PANC-1 cells (*in vitro*)	(26)
Rapamycin ± gemcitabine	Kras PTEN-deficient mice model (Pdx1-Cre, KrasG12D/+, Ptenflox/+, *in vivo*)	(27)
AZD8055 ± erlotinib	PANC-1 and Capan-1 cells (*in vitro*); mouse PANC-1 xenografts (*in vivo*)	(3)
Rapamycin ± XRT (4 Gy)	PC-2 and PANC-1 cells (*in vitro*)	(28)
INK128 ± XRT	PSN1, MIA PaCa-2, and PANC-1 cells (*in vitro*), mouse PSN1 xenografts (*in vivo*)	(29)
Rapamycin ± metformin	Mouse Panc02 xenografts (*in vivo*)	(30)
AZD8055 + BEZ235	Kras p53-inactivated mice model (Pdx1-Cre, LSL-KRASG12D, p53Lox/+, *in vivo*)	(31)

*INK-128, mTORC1/2 inhibitor; PD0325901, MEK inhibitor; BEZ235, dual PI3K/mTOR kinase inhibitor; PKI-587, dual PI3K/mTOR kinase inhibitor; GDC-0980, dual PI3K/mTOR kinase inhibitor; AZD8055, mTORC1/2 inhibitor; XRT, radiation therapy; Gy, gray*.

Mammalian target of rapamycin inhibition with first and second generation inhibitors also leads to class specific mechanisms of resistance. As demonstrated across several cancer cell lines, resistance to mTOR-related cytotoxicity has been limited by feedback activation *via* the IGF-1R–AKT signaling pathway imparting drug resistance (32). In PANC-1 and MiaPaCa-2 cells, rapamycin treatment leads to resistance mediated by AKT phosphorylation despite inhibitory effects on proliferation of PANC-1 cells (24). Furthermore, treatment with second generation mTOR inhibitors, such as KU63794 and PP242, leads to treatment resistance *via* increased ERK activation (26).

Novel mTOR inhibitors may overcome resistance mechanisms by dual inhibition of mTOR complexes. Primary and transformed pancreatic cancer cells exhibit a concentration- and time-dependent arrest of growth upon dual mTOR inhibition with INK-128 *via* 4E-BP1, S6K1, and AKT (25). Furthermore, INK-128 also sensitizes cells to treatment with gemcitabine. Use of U126 or PD0325901 MEK inhibitors prevents ERK overactivation induced by NPV-BEZ235 (dual PI3K/mTOR kinase inhibitor) leading to synergistic inhibition of proliferation in a dose-dependent manner in PANC-1 and MiaPaCa-2 cells (26).

### *In Vivo* mTOR Inhibition Studies

Animal models have demonstrated that agents targeting the mTOR pathway can lead to significant inhibition of proliferation, differentiation, and tumor progression in specific PDAC subpopulations (Table 1). Enhanced inhibition of tumor differentiation and progression by rapamycin was demonstrated to be specifically dependent on loss of PTEN in KRAS-mutant mice (KC) (27). Inhibition of mTOR improved survival and induced tumor shrinkage downstream of mTOR *via* S6 leading to regression of tumors into benign, relatively non-proliferative cysts. In contrast, KRAS-mutant mice tumors driven by mutant p53 (KPC) did not respond to rapamycin treatment, which has other distinct pathways that are mTOR independent (27, 33). In transgenic mouse models in which mTOR was hyperactivated either through the KRAS/MEK/ERK cascade, by loss of PTEN, or through TSC1 haploinsufficiency, single inhibition of mTOR or MEK elicited strong feedback activation of ERK or AKT (34). In this study, rapamycin and PD98059 individually lead to ERK and AKT feedback-mediated resistance; however, dual inhibition with LY294002 and PD98059 ameliorated oncogenic activity. Furthermore, PTEN-deficient cells responded to LY294002 and/or rapamycin treatment, but not PD98059, consistent with aforementioned study by Morran and colleagues (27). Analysis of downstream targets in pancreatic cancer cell lines identified that MEK/ERK/TSC/mTOR signaling is dependent on ALDH1A3 function and high expression of ALDH1A3 is associated with an aggressive subtype of PDAC (34). Therefore, in ALDH1A3-positive PDAC, targeting of ALDH1A3 may be of benefit in addition to inhibiting the MEK/ERK/mTOR cascade. Moreover, PTEN haploinsufficiency also appears to promote tumorigenesis through PI3K-dependent NF-κB activation in pancreatic cancer mouse models (35). Notably, treatment with LY294002 abrogated NF-κB activation in PTEN haploinsufficient pancreatic cancer models *in vivo* (35).

Use of second generation mTOR inhibitors offers similar distinct mechanisms of tumorigenesis inhibition. AZD8055, a second generation mTOR inhibitor, used with erlotinib (an EGRF inhibitor) leads to proliferative inhibition in PANC-1 xenografts (3). Use of both AZD8055 and erlotinib abolished EGFR/AKT feedback activation-related resistance associated with AZD8055 monotherapy. The combination of AZD8055 and the dual PI3K-mTOR inhibitor BEZ235 delayed PDAC progression and prolonged survival in KRAS-mutant PDAC mice *in vivo* (31).

### mTOR Inhibitors as Radiosensitizers

First and second generation mTOR inhibitors both act to sensitize PDAC to radiation therapy *in vitro* (Table 1). PC-2 and PANC-1 cells treated with rapamycin exhibited a dose-dependent radiosensitizing effect on cell proliferation arrest leading to G2/M phase cell cycle arrest (28). PSN1 cells exhibited a dose-dependent inhibition of proliferation and tumor growth delay in athymic nude mice xenografts following single and fractionated doses of radiation with INK-128 pretreatment (29).

### Metformin-Related mTOR Inhibition of PDAC

Metformin exhibits diverse effects on PDAC carcinogenesis through both mTOR-dependent and -independent mechanisms (36). Metformin mTOR activation occurs *via* AMPK-mediated (16, 37), Rag GTPase-mediated (17), and REDD1-mediated mechanisms (18). Metformin may also ameliorate aberrant signaling and feedback inhibition *via* insulin-like growth factor-1 receptor (IGF-1R)–AKT signaling by improving insulin tissue sensitivity (16). In MIAPaca2 and PANC1 cells explanted into an athymic nude mice xenograft, metformin inhibits pancreatic cell growth *via* mTOR1 inhibition, which was demonstrated to be dose dependent (38). Combined treatment with metformin and rapamycin of Panc02 cells transplanted into diet-induced obese (DIO) C57BL/6 mice lead to significantly reduced pancreatic tumor growth and mTOR-related signaling (30).

### Inhibition of mTOR in Human PDAC

In genetic profiles of human tumors, loss of or low PTEN expression and hyperphosphorylation of AKT has been found to be present in around 70% of the cases and PTEN genomic loss (deletion of one or two copies of the PTEN locus) in 15% of the cases (35). Even single-copy PTEN loss, in the setting of KRAS-initiated pancreatic transformation, retains its tumorigenic potential by increasing epithelial proliferation, contributing to an aggressive histologic phenotype, and activating PI3K/AKT and ERK signaling *in vivo* (35). Biopsies of human pancreatic cancer cells obtained through endoscopic ultrasonography that overexpressed pS6 (a downsteam effector activated by mTOR overexpression) show sensitivity to ­rapamycin inhibition *ex vivo* (21). Recent data examining resected PDAC biology utilizing “Multi-Omic” analysis suggest that alterations in mTOR pathway are very common and potentially important for treatment (39–42). Out of 117 PDAC samples included in one analysis, 43% of the patients had a therapeutic response related to a molecular abnormality or mechanism/pathway identified through next-generation sequencing (NGS) (41). Actionable findings linked to a specific treatment options identified by NGS included mutations in BRCA2 (5%), PALB2 (1%), ATM (4%), BRAF (2%), PIK3C/PIK3R (7%), STK11 (5%), amplification of ERBB2 (3%), FGFR (2%), PDGFR (2%), and RET fusions (2%) (42). Further analysis with incorporation of immunohistochemistry (IHC) in this cohort refined and expanded chemotherapy treatment options in all patients (41). A subset of PDAC with integration of phosphoproteomics (PHO) in their NGS and IHC analysis revealed pathway activation (e.g., mTOR, JAK-STAT, MET, RET, or EGFR) in 16/20 samples (41).

Unique genetically driven human PDAC have been reported to respond to targeted mTOR inhibition suggesting that preclinical data are applicable in the treatment of human PDAC. During phase I treatment with MK-2206, a pan-AKT inhibitor, a dramatic 23% shrinkage in tumor was found in a patient with a loss of PTEN KRAS-dependent PDAC, which was thought to have occurred *via* PI3K–AKT–mTOR inhibition (43). Similarly, use of everolimus in a patient with Peutz–Jeghers syndrome-induced advanced pancreatic cancer with presumed mTOR hyperactivation through loss of STK11/LKB1 leads to 9 months progression-free survival (44).

## Clinical Trials

### Clinical Trials Utilizing mTOR Inhibitors

Rapalogs monotherapy have not been shown to be effective in three phase II clinical trials of gemcitabine-refractory metastatic PDAC. Although treatment was well tolerated in a multi-institutional, single-arm, phase II study of everolimus in patients with gemcitabine-refractory metastatic PDAC, there were no significant improvements in progression-free survival or overall survival (45). The most common grade 3 and 4 treatment-related toxicities were thrombocytopenia and hyperglycemia, respectively, leading to delay in treatment; however, no patients were removed from treatment due to drug-related adverse effects. In an open label, single-arm phase II study in gemcitabine-refractory metastatic PDAC patients treated with either temsirolimus or everolimus/erlotinib, there was similarly no demonstrated improvement in clinical responses (46). Significant systemic toxicity leads to premature cessation of patient enrollment for patients treated with temsirolimus. Treatment with everolimus/erlotinib therapy was tolerated; however, enrollment was also prematurely ended due to progression of disease on therapy.

Although not a direct mTOR inhibitor, MK-2206, an allosteric AKT inhibitor, with selumetinib, a MEK1/MEK2 inhibitor, were included in phase II trial concerning metastatic PDAC failing gemcitabine-based therapy. Patients were randomized to treatment groups MK-2206 135 mg weekly plus selumetinib 100 mg daily (MS) or mFOLFOX6 (without 5-FU bolus) every 2 weeks. The most common toxicities, including rash, mucositis, dehydration, and fatigue, were observed in 34 patients in the MS arm compared to hematologic toxicities, fatigue, nausea, and vomiting observed in 19 patients in the mFOLFOX arm. MS did not improve overall survival, and shorter survival was observed compared to mFOLFOX [median OS 4.0 vs. 7.5 months, hazard ratio (HR) 1.46, 95% CI 0.90–2.38] (47).

Recently, combination therapy with capecitabine (5-FU prodrug) and everolimus in a phase II trial of the first-line and second-line treatment of PDAC demonstrated modest benefit to combined therapy over monotherapy (48). Median overall survival with combination therapy was 12.4 months in first-line patients and 5.9 months in second-line patients compared to historical rates of overall survival with capecitabine monotherapy of 5.9 months in first-line patients and 5.0 months in second-line patients suggesting that addition of everolimus to capecitabine might enhance efficacy of capecitabine monotherapy, especially in first-line patients.

Limitations of the aforementioned trials include the lack of characterization of the molecular pathology underlying PDAC tumorigenesis, which preclinical data suggest would predict treatment response. The modest improvement noted in the combined therapy everolimus/capecitabine phase II study supports the notion that coordinated inhibition of downstream KRAS signaling may improve antitumor efficacy of therapy (NCT01337765, NCT01324258, and NCT01562899).

## Conclusion

Although initial trials targeting mTOR inhibition have generally failed to demonstrate treatment efficacy, specifically targeting therapy to the diverse mutations underlying KRAS-dependent PDAC in specific subpopulations offer potential for coordinated inhibition of synergistic trophic mechanisms and the resistance-related feedback mechanisms underlying tumorigenesis. Targeting these mutations offers the advantage of improved tumor treatment with the potential for less systemic toxicity. Inhibition of patient-specific mTOR activity has strong preclinical data. Although many initial clinical trials with mTOR inhibition have been negative, the coordinated inhibition of multiple steps along the mTOR pathway may offer a viable alternative form of treatment for genetically defined PDAC in the future. Results from further late-phase studies involving combined mTOR pathway inhibition are eagerly awaited.

## Author Contributions

SI and SA are co-first authors of this paper as they contributed to concept, initial drafting, and literature review. All authors otherwise contributed to this paper through literature review, drafting, critical revision, editing, and final approval of the final version.

## Conflict of Interest Statement

The authors declare that the research was conducted in the absence of any commercial or financial relationships that could be construed as a potential conflict of interest.
